# Evidence-Based Health Care Policy in Reimbursement Decisions: Lessons from a Series of Six Equivocal Case-Studies

**DOI:** 10.1371/journal.pone.0078662

**Published:** 2013-10-30

**Authors:** Pieter Van Herck, Lieven Annemans, Walter Sermeus, Dirk Ramaekers

**Affiliations:** 1 Center for Health Services and Nursing Research, University of Leuven, Leuven, Belgium; 2 Department of Public Health, I-CHER, Ghent University, Ghent, Belgium; Groningen Research Institute of Pharmacy, The Netherlands

## Abstract

**Context:**

Health care technological evolution through new drugs, implants and other interventions is a key driver of healthcare spending. Policy makers are currently challenged to strengthen the evidence for and cost-effectiveness of reimbursement decisions, while not reducing the capacity for real innovations. This article examines six cases of reimbursement decision making at the national health insurance authority in Belgium, with outcomes that were contested from an evidence-based perspective in scientific or public media.

**Methods:**

In depth interviews with key stakeholders based on the adapted framework of Davies allowed us to identify the relative impact of clinical and health economic evidence; experience, expertise & judgment; financial impact & resources; values, ideology & political beliefs; habit & tradition; lobbyists & pressure groups; pragmatics & contingencies; media attention; and adoption from other payers & countries.

**Findings:**

Evidence was not the sole criterion on which reimbursement decisions were based. Across six equivocal cases numerous other criteria were perceived to influence reimbursement policy. These included other considerations that stakeholders deemed crucial in this area, such as taking into account the cost to the patient, and managing crisis scenarios. However, negative impacts were also reported, in the form of bypassing regular procedures unnecessarily, dominance of an opinion leader, using information selectively, and influential conflicts of interest.

**Conclusions:**

‘Evidence’ and ‘negotiation’ are both essential inputs of reimbursement policy. Yet, purposely selected equivocal cases in Belgium provide a rich source to learn from and to improve the interaction between both. We formulated policy recommendations to reconcile the impact of all factors identified. A more systematic approach to reimburse new care may be one of many instruments to resolve the budgetary crisis in health care in other countries as well, by separating what is truly innovative and value for money from additional ‘waste’.

## Introduction

Innovation of clinical practice has enabled professional care providers to improve population health in a way that would have been impossible before the 21th century. Yet, ironically, the same drive for innovation and the related technology push from industry is currently often cited as one of the main reasons why health systems across Western countries are spinning out of control [1]. Spending levels are rising well above 10% of gross domestic product, and in times of diminished economic growth the current expenditure growth cannot be sustained [2]. Across-the-board cuts are looming, with unpredictable consequences for the quality of care.

There is a growing consensus that the rise in spending should be topped off at the level justified by demographic changes and inflation. This, however, might threaten the future capacity for innovation. New drugs, implants and other care interventions enter the market continuously. Nowadays policy makers are faced with the increasingly difficult challenge to decide which new entrants will be reimbursed and which will not. The general public often presumes that this decision making process is done in a systematic manner, guided by evidence of (cost) effectiveness. Evidence based policy implies that policy makers make well informed decisions about policies, programs and projects by putting the best available evidence from research at the heart of policy development and implementation [3]. In our context, evidence includes both clinical and economic knowledge from research about (cost) effectiveness to support or negate the appropriateness of reimbursement. This is further specified as (1) therapeutic added value compared to existing therapeutic alternatives in terms of mortality, morbidity and/or quality of life, and (2) the ratio of cost to society per unit of therapeutic value. Yet, reimbursement policy involves negotiation between payers (state and/or insurers), care providers (professional interest groups) and, sometimes, suppliers (pharmaceutical industry and manufacturers)[4].

**Figure 1:**
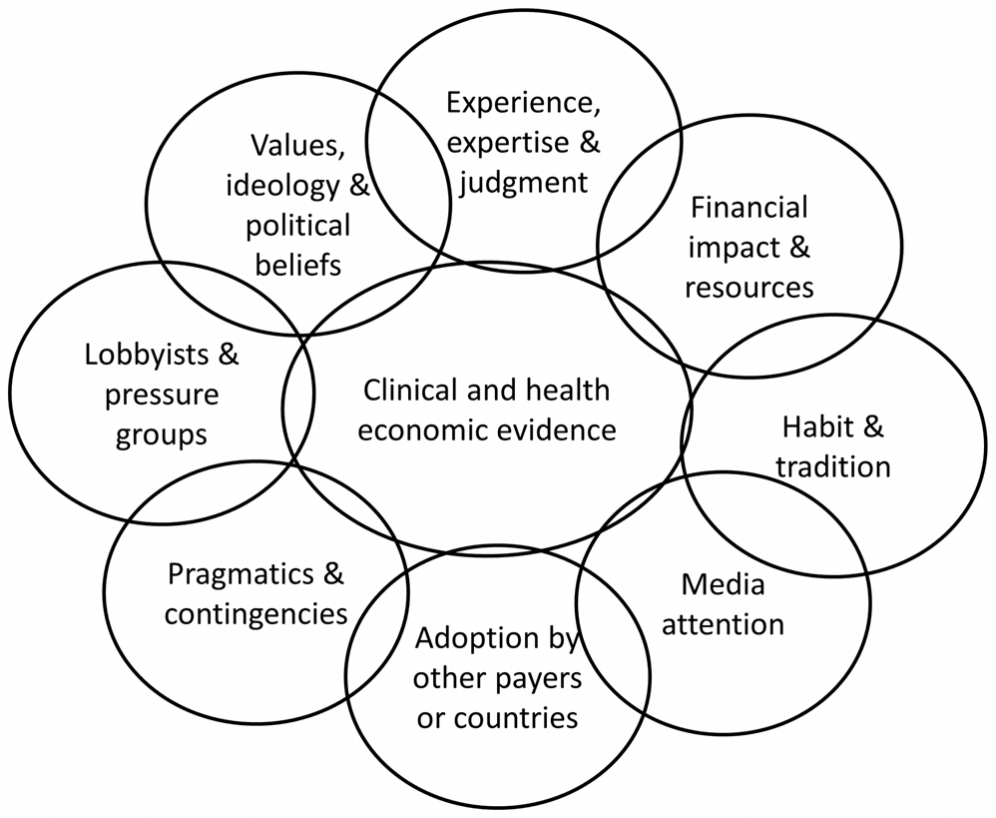
Evidence based policy framework. * Legend: *Partially adapted from Philip Davies (2004), [3].

According to the evidence based policy framework formulated by Philip Davies, the decision to include or exclude an innovation in/from reimbursement will, next to clinical and health economic evidence, theoretically also depend on (1) Experience, expertise and judgment of decision makers, (2) Financial impact – including cost-effectiveness – and resources, (3) Values, ideology and political beliefs, (4) Habit and tradition, (5) Lobbyists and pressure groups, and (6) Pragmatics and contingencies [3]. In times of hypes and trends in adopting care innovations [5], we add (7) Media attention and (8) Adoption of innovation by other payers or countries to this framework (see Figure 1). We elaborate further on these concepts in Table 1.

**Table 1:** Concepts of the evidence based policy framework explained.*

**Clinical and health economic evidence:** Evidence helps the stakeholders make well informed decisions about reimbursement of new drugs, medical products and care interventions by putting the best available knowledge from research at the heart of policy development and implementation. The stronger the evidence for effectiveness, and thus potential gain in population health, the stronger the case for reimbursing an innovative diagnosis or treatment intervention.
**Experience, expertise & judgment:** Stakeholders bring a considerable degree of human and intellectual capital to the table, also known as tacit knowledge. Judgment based on experience and expertise is especially critical for decisions where evidence is lacking or needs further nuanced interpretation due to limitations or inconsistencies. The latter is often the case in reimbursement decision making. In real life policy making without the impact of experience, expertise & judgment is impossible.
**Financial impact & resources:** In a world of limited (and declining) resources, a reimbursement decision also takes into account the cost to society by comparing innovations and status quo based on cost per unit of health gain, within available budget limitations. Ideally this is done by cost-effectiveness, cost-benefit or cost-utility analysis. In addition, the cost to the individual patient is also taken into account to safeguard financial accessibility as a determining factor of equity of care.
**Values, ideology and political beliefs:** Even when the effects or absence of effects of a new care intervention have been empirically demonstrated, values, ideology and political beliefs remain a major driving force of policy making. As Philip Davies notes, the tension between both is at the centre of contemporary politics in open democratic societies, also in terms of reimbursement decision making.
**Habit & tradition:** Reimbursement policies in each health system have a history of the way in which decisions are made, and factors taken into account when making those decisions. Habits are often locked into and reinforced by formal and informal procedures. Aligning those parts of the system with new insights into how reimbursement policies could rationally be improved represents a continuous challenge.
**Lobbyists & pressure groups:** Lobbyist and pressure groups seek to optimize interests and power relations external to what is best for the patient according to evidence. The latter is therefore often used in a less systematic and selective way based on the (mis)match between evidence and interests.
**Pragmatics & contingencies:** An inherent constraint of the reimbursement decision making process are the short vs. long term time table, with evidence accruing only slowly. In a context of unanticipated contingencies such as an epidemic outbreak or other public health crisis, policy often has to move forward at a speed that does not allow the time to be thoroughly informed by evidence.
**Media attention:** The media are another potential influence on reimbursement decision making. Like lobbyists and pressure groups, the media often make use of evidence in a less systematic and selective way. The media influence public opinion about what should and what should not be reimbursed. Both payers and care providers might be sensitive to this.
**Adoption by other payers or countries:** Payers, being governments, health funds or insurers, do not decide on reimbursement in isolation. Decisions of governments are expected to be influenced by what happens across country or state borders. Similarly, insurers take into account what is reimbursed or planned to be reimbursed by their competitors. Care provider representatives will also look across borders to state their case.

* Partially adapted from Philip Davies (2004), [3].

In real life reimbursement policy, the relative weight of each of these factors on the final outcome of decision making, and how these factors interact, are both not clear [3]–[4]. The aim of this study is to explore this complex interplay in a series of six case-studies. The study focused on the following research questions: (1) What was the relative impact of each of the framework factors on the decisions made in six case-studies, as perceived by key stakeholders? (2) How did such factors influence systematic decision making, expected to be guided by evidence? And (3) which pattern, if any, of differences and similarities of impact can be identified across six case-studies?

## Methods

### Ethics statement

The study was approved by the University of Leuven ethical review board, sub domain of public health. Written informed consent was obtained from all participants.

Stakeholders included key decision makers directly involved in the decision to approve or decline reimbursement at a national level in one or more of the six case-studies. The scope of our study is restricted to the Belgian health system, wherein negotiation occurs between federal government and sickness funds as payers, and physician, and pharmacy representatives as care providers, completed with academics. In Belgium, reimbursement decisions for drugs and implants are guided by separate directives of the EU that specify adoption requirements. Decision making takes place in three national councils of the above representatives, as part of the responsibility of the National Institute of Health and Disability Insurance. One council makes reimbursement decisions about medical drugs, another about medical devices such as implants, and a third about medical fees for services unrelated to a specific drug or device (such as breast cancer screening).

An overall set up of negotiation is considered to be typical for part of the Western health systems, as illustrated in many health system profiles, published by the Health Systems & Policies Observatory of the World Health Organization [6].

The study made use of a series of six qualitative case-studies. Cases, being national reimbursement decisions on new drugs, implants or other care interventions that are known to be contestable from a purely clinical and health economic evidence-based perspective, were selected in the following way: First, both media publications and scientific literature were screened to devise a list of candidate cases, based on expressed doubt about the evidence-based nature of reimbursement decisions made. Secondly, the research team selected six cases that covered drugs, implants and other medical services in a balanced way. The case studies address aortic endovascular replacement, breast cancer screening, oseltamivir, hadron therapy, Alzheimer medication, and trastuzumab. We emphasize that the selection of these cases was not based on a fully-agreed upon lack of evidence; nor was it the result of the subjective opinion of ourselves as investigators. Literature revealed that in the selected cases inconsistencies of study findings or discrepancies of normal procedures were likely at play. Cases were focused upon to learn from, without judgment or allocating blame.

Data were collected through in depth semi structured interviews of eight key stakeholders, who were recruited based on their involvement in cases and their central role in the decision making process. At least two stakeholders were interviewed per case. In Table 2 the stakeholders’ role in cases is presented. The interview protocol was constructed according to the evidence based policy framework as presented in Figure 1, with additional content validity testing and piloting by three experts. The general interview guide approach was used, providing both structure and flexibility by adapting questions to the individual interview process. It ensured that the same general areas of information were collected from each participant, but still allowed a necessary degree of freedom and adaptability. All data were collected by the same interviewer who was trained beforehand by means of pre-interview exercises and a pilot test. The purpose, format and confidentiality were explained to each participant. Questions were phrased in a neutral and mostly open-ended format. State-of-the-art guidelines in qualitative data collection were followed [7]-[8]. The duration of the interviews varied from 48 minutes up to 1 h 15 minutes. Interviews were tape recorded and transcribed in full. Participants had the opportunity to revise the transcript for further clarification.

**Table 2:** The professional role of participating stakeholders in cases.

	Aorta endoprosthesis	Breast cancer screening	Oseltamivir	Hadron therapy	Alzheimer medication	Trastuzumab
Respondent 1: Physician and politician		X	X			
Respondent 2: Health fund	X		X		X	X
Respondent 3: Health fund	X			X		
Respondent 4: Physician		X				
Respondent 5: Physician and expert			X	X		
Respondent 6: Physician	X					
Respondent 7: Government						X
Respondent 8: Government					X	

Thematic analysis was applied: First, themes were extracted according to the categories of the evidence based policy framework. Secondly, relevant text fragments were allocated to themes. We also sought to cover additional themes, if present, which were not addressed in the framework. Thematic analysis was performed by HT, and independently validated by PVH and DR. Disagreement was resolved through discussion until consensus was reached.

## Results

In this section we first describe the specific context of each case. After separately reporting the results for six cases on the stakeholder perceived impact of each factor, we cross our findings across cases to describe relevant patterns. We use the following codes to label cited text fragments according to stakeholder role: CP  =  care provider/physician; G  =  government; and HF  =  health fund/insurer.

### The aortic endovascular replacement (EVAR) case

#### Context

In the year 2000, while evidence for this procedure was still lacking, payers in Belgium decided to reimburse aortic endovascular replacement, based on the presumption that this would decrease the need for open surgery with its associated potential complications. A reimbursement procedure meant to temporarily support experimental testing of new techniques in a limited number of hospitals, was extended to the majority of all Belgian hospitals. However, in 2005 mounting evidence – including the EVAR I and II RCT in the UK – showed that the gain of less complications and mortality may be present during 1 to 2 years only, after which this benefit disappears [9]–[10]. In the EVAR II group with inoperable patients, there was no mortality benefit versus no intervention at all. In addition, many additional complications, stent dislocation, leakages, and need for revisions were the consequence of aorta endoprosthesis introduction, at a rate of fivefold more complications. This resulted into an up to 20% reintervention rate and the need for lifelong surveillance. Although at present long term cost-effectiveness is not clear, in short term (up to four years) EVAR is more expensive than open surgery, with a mean hospital cost difference per patient of 3311£ for low risk cases and 8649£ for high risk cases in UK trials, presenting the strongest evidence [11]–[12].

#### Clinical and health economic evidence

All three respondents recognized that at the time of ‘experimental’ reimbursement insufficient evidence was available. Respondent 3 (HF) sees this as a typical example of a case in care for which evidence is expected to develop later on, as an argument for this kind of temporary reimbursement. In those days (year 2000) evidence was not sought systematically (Resp. 3, HF, and 6, CP). The supplier firm hired vascular surgeons and paid them to demonstrate the technique to colleagues (Resp. 3, HF). This kind of ‘tutoring’ spread wildly across a too high number of centers, with as a result some centers placing only two or three stents a year (Resp. 6, CP). According to resp. 6 (CP) some expert opinion leaders in vascular surgery claimed that there was evidence, a statement that as such was taken into account in the reimbursement decision. All three respondents agreed that the growing use of this procedure in practice, despite a lack of evidence, made it very difficult not to reimburse. ‘Regulation was caught up by the facts’ as resp. 6 (CP) noted. A lot of stents were already being placed.

#### Financial impact & resources

Cost-effectiveness was not considered, only the additional cost of the new procedure as such: higher prosthesis costs compensated by a lower length of stay (resp. 6, CP). Resp. 2 (HF) commented that the high cost of procedure, making it almost financially unaffordable for patients to bear, added to the pressure to reimburse. Resp. 6 (CP) agreed with this. Resp. 3 (HF) added the following observation:

“If a patient entered with an aneurysm, which can become a life threatening condition, one did not check first whether he/she could afford to pay for the procedure. A prosthesis was placed. This was an important argument to grant reimbursement.”

#### Habit & tradition

In this case the regular decision making process was not followed: A shortcut was taken by allowing so called temporary experimental reimbursement to spread via a convention with hospitals. Respondent 6 (GP) confirms that such a deviation is not exceptional, even today, whereas respondent 3 (HF) disagrees. In line with the external pressure, additional speed lies often at the foundation of the decision to quicken the process. Up to eight months could be gained in this way (resp. 3, HF).

#### Media attention

Before the decision making process even started, a popular newsletter had published an article titled ‘I am sitting here, waiting, with a bomb in my body’ (resp. 3, HF, and 6, CP). It mentioned that the government refused to reimburse the procedure. The article ended up the same day on the desk of the minister of social affairs. Despite many question marks, this hastened the process up to three weeks (resp. 3, HF). The minister was also invited by the media to explain his position (resp. 6, CP).

#### Lobbyists & pressure groups

Resp. 3 (CP) confirms that the implant industry pressured vascular surgeons to use the new procedure. He/she adds that industry and the professional interest group had found each other in this endeavor.

“This was the way of the early days, when a technology reached the market because professional interest groups were contacted by the firms. They said: We have a good new device and we will take you out for dinner, or to an international symposium, if you want to try it out. In this way the device was introduced.”

All three respondents agreed that external pressure was the single most important determinant of the decision to grant reimbursement. Pressure came from care providers, suppliers and patients, the latter mainly due to the cost size for them to bear without reimbursement.

### The breast cancer screening case

#### Context

Mammography is used in both breast cancer screening and in diagnostic case-finding. A screening mammography is intended to detect a tumor in the pre-clinical stage. The procedure needs to adhere to strict technical quality criteria and a double reading by two trained radiologists. Diagnostic case-finding on the other hand is intended to be applied if a patient has complaints or if a care provider has detected an anomaly on clinical examination. The quality of this last procedure is less guaranteed than for a screening mammography. If a diagnostic mammography is used as a first screening tool, the risk of detecting false positives will increase [13]. This type of inappropriateness increases the emotional burden of patients and cost to society in a way that can be avoided to a larger degree with systematic screening mammography use. Cost-effectiveness studies of screening mammography, assuming that mortality was reduced with 30%, predicted a 16000$ cost per year of life expectancy saved for women aged 50 to 69 years [14]–[15]. In Belgium since 2001 both procedures are reimbursed, independently of their indication. A large amount of inappropriate diagnostic screening has been observed in Belgium, with significant geographical variability [16].

#### Clinical and health economic evidence

Evidence played a crucial part in the decision to reimburse screening mammography. Screening was promoted based on the evidence (resp. 1, CP/G). Yet, respondent 4 (CP) adds that the evidence is currently the topic of some heated debates, because Belgium does not reach a sufficient screening coverage of the population to reach optimal results. “If, after ten years, such conditions are not met, you lose a large part of the evidence based potential.” (resp. 4, CP)

#### Experience, expertise & judgment

Both respondents (CP/G) agreed that the initiative in this case mainly came from the government and policy makers themselves. A broad input of expertise has been taken into account (scientific associations, universities, local communities, health funds) (resp. 1, CP/G).

#### Habit & tradition

The decision to organize screening mammography at a population level and not only after referral by a physician, implied a change from traditions and developing new habits, although this met large resistance of some health care professionals (resp. 1 CP/G). Resp. 1 also mentioned the habits of the patient population as a restraining factor to organize screening more systematically and efficiently: ‘As a patient you go to a physician, you get what you want. You want to have a mammography right away, because you are worried’. Patients do not always follow the rules and regulations. Making patients more responsible in this matter remains a challenge. Respondent 4 (CP) confirms that neither the care provider nor the patient are motivated “to follow the correct way”.

#### Adoption by other payers or countries

The fact that neighboring countries already applied screening mammography boosted public demand, which in turn incited policy makers to take initiative (resp. 4, CP). Adoption by other countries was interpreted by some decision makers as a proof of evidence (resp. 4, CP). The way in which screening was subsequently organized, did however not correspond with the approach used in other countries, such as the Netherlands and Scandinavia, where the screening process was more steered by the state (resp. 1, CP/G, resp. 4, CP).

#### Pragmatics & contingencies

Jurisdictions of regional versus federal government complicated and delayed matters. Prevention is a jurisdiction of the regions in Belgium, whereas the financing partly had to come from the federal level. Regional responsibility is also perceived to be related to currently observed geographical variability in the use of screening mammography versus diagnostic screening (resp. 4, CP).

#### Lobbyists & pressure groups

According to resp. 1 (CP/G), pressure from the medical corps and from radiologists in particular resulted into a large degree of therapeutic freedom in the choice of screening modalities.

#### Values, ideology & political beliefs

Resp. 1 (CP/G) reported the influence of cultural differences between the north and the south: the north acting in an Anglo-Saxon style by supporting guidelines, the south focusing on therapeutic freedom as is more the tradition in the south of Europe. In line with this, resp. 4 mentioned the physician interpretation of the decision to involve non-physicians in screening as an ideological choice. He said this as follows: “Physicians thought this choice went towards a form of state driven medicine, a medicine of the masses, without personal contact with the physician”. Both respondents make a distinction between political preferences: socialist parties were proponent of a more directed form of medicine, whereas liberal parties were proponent of a more liberal (and freedom affirmative) medicine.

### The Oseltamivir case

#### Context

Oseltamivir is an antiviral drug promoted for both therapeutic and profylactic use in influenza. The drug was massively distributed and prescribed in Belgium during the Mexican flu pandemic in 2009. The evidence for the effectiveness of Oseltamivir is however sparse. The findings of the main supportive study for its use, conducted by its pharmaceutical supplier, have been heavily questioned in top ranking medical journals. Whereas proponents argued that Oseltamivir prevented infection in 80% of contacts [17], decreased mortality with 68 to 91% [18] (based on unpublished data) and decreased lower respiratory tract complications with 55% [19], opponents only accepted the evidence as sufficiently strong for a reduction of duration of illness with about 1.5 day [20]. Belgian authorities supported the use of Oseltamivir at a scale never seen before, and hoarded up massive reserves.

#### Clinical and health economic evidence

Resp. 1 (CP/G), resp. 2 (HF) and resp. 5 (CP) all agree that most decision makers were aware of, and regretted, that clear cut evidence was lacking. It was however difficult to question the purchasing decision, because a primary opinion leader, as a scientist, confirmed the need for providing Oseltamivir freely to high risk patients with prescription and building up sufficient stock, although this point of view was not shared by the scientific community as a whole. “If evidence is lacking, as a politician you listen to the most renowned expert” (resp. 1, CP/G).

#### Experience, expertise & judgment

Resp. 1 (CP/G) wondered whether putting both the scientific and policy responsibility in the hands of one person was not counterproductive. He/she had preferred a clearer cut division of scientific versus policy stakeholder ship, respecting checks and balances. “In this case politicians hid behind the broad back of the scientific expert” (resp. 1).

#### Financial impact & resources

In the heat of emotions, the financial impact was not taken into account (resp. 1, CP/G). According to resp. 2 (HF), in terms of cost-effectiveness this as such represented a disaster. Belgium spent more resources on the purchase than other countries (resp. 5, CP).

#### Habit & tradition

Due to the urgent situation, the regular scientific evaluation by an independent evidence-based drug review was not performed, and instead replaced by the opinion of a few experts (resp. 1, CP/G).

#### Media attention

The media increased the pressure of public opinion, by instilling fear and reinforcing the – real or artificial – urgency of decision making (resp. 1, CP/G).

#### Adoption by other payers or countries

Collaboration and consensus building between countries did not work appropriately. Each country implemented its own policy (Resp. 1, CP/G). In the end, many countries stocked up Oseltamivir. Yet, Belgium did this at the double rate of most other countries, to be completely independent from others (resp. 5, CP).

#### Lobbyists & pressure groups

Public opinion, fearing a flu pandemic, put a large pressure on politicians to do something, even if the true effect was minimal (resp. 2, HF). Resp. 1 (CP/G) mentioned the negative impact of the pharmaceutical industry, that tried to sell products as much as possible, even when little arguments were available.

### The Hadron therapy case

#### Context

Hadron therapy uses protons and neutrons to radiate tumors. Theoretically, this would lead to fewer side effects than commonly used electron and foton-based radiotherapy, especially for children. At present, these assumptions are however not yet been confirmed by clinical trials. Two systematic reviews concluded that no definitive conclusions can be drawn for head and neck cancer, gastrointestinal tumors, non small-cell lung cancer, sarcomas, cancer of the uterine cervix, prostate cancer, most CNS tumors, and bladder cancer [21]–[22]. The number of current indications for the use of Hadron therapy is very limited, with only some preliminary case studies on clinical benefit for rare ocular melanomas and base of skull chordomas (51 patients per year in Belgium). Despite scientifically founded independent advice not to do so, a working group of all radiotherapy centers in Belgian teaching hospitals have received green light to prepare the building of a hadron center. If introduced, this will represent an investment cost of 159 million euro, taking up a substantial proportion of the planned budget to fight cancer [21]–[22].

#### Clinical and health economic evidence

The respondents (3, HF and 5, CP) indicate that this decision is being prepared with ups and downs in the degree to which the evidence is taken into account. At first, the evidence was put to the centre of the decision. However, in a second term of legislation, with a new minister on board, the evidence has moved to the background and the decision is more ‘politicized’. Previous studies are ignored, and under the pretence of doing a new study, reimbursement and implementation are being prepared.

#### Financial impact & resources

Despite knowledge of lacking cost-effectiveness, the ministry pursues its plans (resp. 3, HF and 5, CP). Positive financial impact of hadron therapy as a regional investment strategy, for care providers and the local population to benefit from, and for politicians, to defend local voters, has surpassed patient value as one of the most determining factors (resp. 3, HF and 5, CP).

#### Habit & tradition

Evaluation procedures were followed at first. However, when politicians disagreed with the outcome, regular procedures were being shortcut by an additional study with external budgets controlled by the ministry itself, and not by the normal stakeholder groups at government level (resp. 3, HF).

Resp. 3 (HF) also mentioned the habit in health care of wanting to implement everything everywhere, because of the level of prestige associated. Only the investment cost has prevented the development of likely six to seven hadron centra across a small country.

#### Adoption by other payers or countries

Larger countries have a sufficient number of patients eligible for this type of care. This is, however, not the case in Belgium (both resp.).

#### Lobbyists & pressure groups

Respondents mentioned that radiotherapy centers of teaching hospitals, which represent both the scientific expertise, and the potential gainer of a decision to reimburse and implement, have no unbiased role in this case (resp. 3, HF). This is one of the reasons that this case is repeatedly put onto the table, despite lacking evidence. In addition, local industry acted as lobbyist to attract additional investments (resp. 5, CP).

#### Values, ideology & political beliefs

Politics dominated in this case. One of the respondents referred to “an angry crowd of politicians in power who disagree with scientific recommendations” (resp. 3, HF).

### The Alzheimer medication case

#### Context

Dementia drugs, such as inhibitors of acetylcholinesterase (ChEIs) and memantine, are used to temporarily alleviate the symptoms of the disease. Both types were reimbursed according to severity of cognitive dysfunction in Belgium since 2002 and 2004, respectively. The clinical and health economic evidence showed mixed results at that time. Due to small effect sizes, the clinical relevance of positive findings was questionable from the onset [23]. Afterwards the number of studies doubled and about a dozen systematic reviews and meta-analyses were published (see [24] for a recent example). Hulstaert and colleagues concluded that dementia drugs improve cognitive functioning with about 1 to 1.5 points on a scale of 30 [23]. Cost-effectiveness studies departed from unrealistically larger effect sizes, and were therefore not reliable. Reimbursement rules were revised in 2011, making them more stringent (e.g. no memantine monotherapy). Yet, the severity of cognitive dysfunction threshold was lowered, making more patients eligible for reimbursement of dementia drugs.

#### Clinical and health economic evidence

Respondents (2, HF and 8, G) reported an evolution from little to a larger degree of evidence-based policy in this case. About a decade ago, it was possible to reimburse something for Alzheimer treatment, even with unreliable evidence. The recent, more stringent reimbursement policy takes evidence much more into account.

#### Experience, expertise & judgment

Whereas external expertise, in terms of providing clinical and health economic evidence, was almost irrelevant at the onset of reimbursement, this factor dominated in recent reform (both resp.).

#### Financial impact & resources

Similarly, a lack of cost-effectiveness, and very high prices demanded by the pharmaceutical industry, were no objection to commence reimbursement (resp. 2, HF). Later on, reimbursement adoptions were not based on a complete cost effectiveness analysis, but moved in that direction. In addition, in line with the evidence, an increase in eligibility based on cognitive functioning, was compensated by a demand to suppliers to decrease prices (resp. 8, G).

#### Media attention

This factor was very relevant a decade ago, with impact on decision making (resp. 2, HF). At present, complaints from patient organizations were taken up by the media. This did not influence decision making (resp. 8, G).

#### Adoption by other payers or countries

Initially, this factor was very relevant since Belgium was one of the last countries in Europe to approve reimbursement of anti-Alzheimer drugs (resp. 2, HF). Recent reform was based on a more systematic review of the evidence, in combination with an international policy analysis (resp. 8, G).

#### Lobbyists & pressure groups

Public demand was high at the time of introduction of anti-Alzheimer medication. This played a large part in the reimbursement decision (resp. 2, HF). After recent reform, some stakeholder groups complained about the more stringent indication setting, backed up by pharmaceutical suppliers. They also involved patient organizations in this matter. Yet, after analysis no argument was found to modify reforms (resp. 8, G).

### The Trastuzumab case

#### Context

Women in an early stage of HER2 positive breast cancer (without metastasis), who receive chemotherapy after surgery, live longer if they are treated with Trastuzumab. Two to three year survival increased with 75 to 89%, [25]. This has been confirmed by five clinical trials, of which the HERA study is most known, [26]. See also the systematic review in Nature [27]. To treat 500 women in Belgium on a yearly basis, this would cost 5.17 million euro to society. Cost-effectiveness was confirmed for a pre-chemotherapy regimen during 9 weeks, with resulting net savings in long term [25]. Belgian government bypassed the regular reimbursement decision making process to approve reimbursement more rapidly.

#### Clinical and health economic evidence

Reimbursement was arranged for, even before all evidence was critically reviewed (resp. 2, HF and 7, G). Resp. 7 formulated this as follows: “If evidence is defined from a purely scientific perspective, then it was not yet present. If evidence is defined as women knocking at the door who stated that their physician said this drug would help them, which it also did, subjectively or not, then this other type of evidence was available”.

#### Financial impact & resources

First, the supplier introduced the drug for a narrow group of patients with metastasized breast cancer, with high prices. After the first approval of reimbursement, the supplier broadened the group of eligible patients to early breast cancer, but did not reduce prices, knowing that public pressure would force decision makers to accept (resp. 2, HF). This strategy is typical for pharmaceutical industries, he/she adds. Making this new expensive drug affordable for all eligible patients was also one of the main reasons why this reimbursement was adopted rather quickly (resp. 7, G).

#### Habit & tradition

To speed up the process, normal procedures were not followed. According to resp. 7 (G) this was understandable to cope with public pressure. However, pharmaceutical industries build up such public pressure to commercialize their products as quickly as possible.

#### Media attention

The media increased public pressure, according to both respondents.

#### Adoption by other payers or countries

Examples of reimbursement in other countries heightened public pressure (resp. 7, G). He adds that this international policy analysis was done in a rather selective way, by focusing on countries with reimbursement, and ignoring countries without.

#### Pragmatics & contingencies

The fact that prescriptions were increasing even before reimbursement was arranged for, reinforced the decision (resp. 7, G).

#### Lobbyists & pressure groups

According to resp. 2 (HF) and 7 (G), trastuzumab was considered to be a ‘wonder drug’. The pressure of society, prescribers and suppliers was therefore significant. Decision makers were swamped with expert opinions, some independently, some sponsored by suppliers.

## Results across cases

Table 3 provides a schematic overview of the impact of each framework factor across reimbursement cases. The impact of (1) evidence was perceived as high in one case, and low in three others. Interestingly, the anti-Alzheimer drug case showed that the impact of evidence was perceived to increase over time, whereas the opposite is true for the hadron therapy case. Experience, expertise & judgment (2) shows a pattern of influence that differs according to the impact of evidence. In low impact of evidence cases, the opinion of one expert or one stakeholder group dominated more. In high impact of evidence cases, input was broader, and included external input by multiple experts.

**Table 3:** The impact of framework factors on reimbursement decisions across six cases.*

	Aorta endoprosthesis	Breast cancer screening	Oseltamivir	Hadron therapy	Alzheimer medication	Trastuzumab
Timeline	Year 2000	Year 2001	Year 2009	Year 2005 up to present	Year 2002, 2004 and 2011	Year 2006
Evidence	Low; no study, caught up by the facts	**High; yet full potential not reached**	Low; no consensus of scientific community sought	**High in first legislation**; low in second legislation	Low at first; **high in recent reform**	Low, before availability
Experience, expertise & judgment	**High; opinion making by care providers**	**High; broad internal and external input**	**High; key impact of one scientific expert**	**High; shift from science to politics**	Low at first; **High, more external input in recent reform**	Low; of no importance
Financial impact & resources	Low with respect to cost to society; **High with respect to cost to the patient**	Not known (Disagreement with respect to cost to society)	Low; with respect to cost to society	**High; shift from cost to society to investment opportunity**	Low at first; **High, more taken into account in recent reform**	Low with respect to cost to society; **High with respect to cost to the patient**
Habit & tradition	**High; a shortcut was taken**	**High; a change from traditions vs. provider/patient habits**	**High; regular procedures were not followed**	**High; regular procedures were not followed in the end**	Low; of no importance	**High; a shortcut was taken**
Media attention	**High; adding pressure**	Low; of no importance	**High; adding pressure**	Low; of no importance	**High at first**; low in recent reform despite presence	**High; adding pressure**
Adoption by other payers or countries	Medium; impact, but less determining	**High; seen as proof, yet no adoption of implementation method**	**High; yet no adoption of implementation method**	**High; despite differing volume conditions**	**High; increasing consistency in adoption of implementation method**	**High; with a selective instead of balanced focus**
Pragmatics & contingencies	Low; of no importance	**High; complicated by jurisdictions**	**High; crisis scenario**	Low; of no importance	Low; of no importance	**High; practice preceded regulation**
Lobbyists & pressure groups	**High; adding pressure, conflict of interest**	**High; adding pressure**	**High; adding pressure**	**High; adding pressure, conflict of interest**	**High at first**; low in recent reform despite presence	**High; adding pressure**
Values, ideology & political beliefs	Low, of no importance	**High; political & cultural differences**	Low, of no importance	**High; politics dominate**	Low; of no importance	Low; of no importance

* Factors with a high impact in a particular case are highlighted in bold.

In terms of financial impact & resources (3), with exception of the first phase in the hadron therapy case and the second phase in the Alzheimer drug case, cost to society was not perceived to have a high impact in any case. Cost to the patient did have a high impact in two cases, both in combination with a low impact of evidence. Habit & tradition (4) was interpreted by respondents in two ways: First, upholding reimbursement decision procedures or not. In low impact of evidence cases, procedures were not followed or shortcuts were taken. Secondly, as inducing a change in healthcare traditions as a consequence of a reimbursement decision. The breast cancer screening case showed that healthcare system habits can be changed, according to respondents. Yet, existing habits did temper the extent to which change could be fully leveraged.

Media attention (5) was perceived to have a high impact in four cases, each time combined with a low impact of evidence. The impact of adoption by other payers or countries (6) was in all cases considered to be medium to high. This was used as a source of information to examine the evidence basis and, in the Alzheimer medication case, to decide to implement. In some cases with low impact of evidence, comparisons were made selectively or independently of contextual differences, to pursue the objective that one or more stakeholder groups aimed at.

Pragmatics & contingencies (7) were reported to have a high impact in three forms: decision making in a public crisis situation, deciding after the care intervention was already widely spread across the market, and having to take into account jurisdictions in decision making of multiple payers, states or regions. The impact of lobbyists & pressure groups (8) was high in all cases, except in the second phase of the Alzheimer medication case. In two cases clear conflicts of interest were reported to impact upon the decision. More specifically, care providers, as co-decision makers, were financial benefiters of a positive reimbursement decision, while they served as scientific experts to objectively examine and report the evidence in a balanced way. Finally, values, ideology & political beliefs (9) had a high perceived impact in two cases, in which both political and cultural differences directed decision making.

## Discussion

To the best of our knowledge, this is one of the first studies to analyze in depth which factors were at the basis of reimbursement decisions, for which the evidence was lacking or debatable. Our findings confirm, in line with other publications, that evidence is not the sole criterion on which reimbursement decisions are based [3]–[4]. The results corroborate the assumptions of the evidence based framework formulated by Philip Davies [3]. All nine factors played a part across the six cases we examined. By extensively interviewing a number of key decision makers in each case, in an atmosphere of trust and anonymity, we were able to lift part of the veil of secrecy that often obscures health policy decision making [28].

We agree with Philip Davies that it is impossible to judge in hindsight whether a good or bad decision has been taken [3]. Our findings illustrate the complexity of health care policy. In a democratic society, where multiple stakeholder groups negotiate to reach a ‘relatively best’ decision, factors such as politics and media attention each have their own legitimate place. To state that only evidence and cost-effectiveness should guide reimbursement decisions, runs the risk of introducing a technocratic approach blind to the multiple concerns and trade-offs of current society.

However, the results provide policy makers with conclusions that could guide future reform in reimbursement decision making. Health care across developed countries is in dire need of system improvements that safeguard what works well for the patient– the legitimate impact of many factors we described – and, simultaneously, allow us to cope with ever rising budgetary challenges. Here we formulate nine recommendations for policy makers in Belgium and in other countries that make use of payer-provider reimbursement councils by linking them to the cases in our study.

1. Separate a standard decision procedure from a ‘fast track’ procedure to allow rapid reimbursement of new care interventions with an exceptional value for public health (such as trastuzumab). Use fast track by exception only, based on a fixed set of formalized criteria and with full public transparency. Give no stakeholder group the freedom to decide singularly to deviate from one of both procedures (e.g. hadron therapy).
2. As a first ‘evidence’ stage of both procedures, summarize all available evidence by a full or rapid health technology assessment, providing policy makers with a balanced overview of inconsistencies and remaining gaps in the evidence, with all pro’s and con’s of a positive versus negative decision (all cases). Speed up, but do not discard, this stage as part of the fast track procedure (aorta endoprosthesis, oseltamivir, trastuzumab). Document cost to the health care system (all cases) and cost to the patient (aorta endoprosthesis, trastuzumab) objectively, with cost-effectiveness analysis.
3. During the ‘evidence’ stage, invite an external panel of key experts in the care intervention subject under review to reach consensus. Select members of the panel independently, for example based on the number and quality of contributions to knowledge as authors of peer reviewed papers on the topic, and make their conflicts of interest public. A representation of the scientific community by one or two persons is insufficient (oseltamivir). Because experts often are local care providers (aorta endoprosthesis, hadron therapy), compose the panel with an equally large proportion of experts who will not benefit from any decision outcome. As our cases show, often a professional group as a whole has a financial conflict of interest. Involving international experts, with selection based on publications, could offer a partial solution to address this problem.
4. Evidence should not only address the yes or no decision to reimburse or not. It should also address how to implement a positive reimbursement decision further in practice, based on the methods that have shown most optimal results.
5. Make a country or payer comparison part of the ‘evidence’ stage (including ‘implementation’ evidence), with equal attention to countries or payers who decided to reimburse, as to the ones that decided otherwise (trastuzumab).
6. In times of a public health crisis, time may be lacking for a comprehensive ‘evidence’ stage. Still, consult a sufficient number of local and international scientific experts to inform policy makers in a balanced way (oseltamivir).
7. Although there is a call to involve stakeholder groups even more in policy making, we think that the factors ‘experience, expertise & judgment’, ‘values, ideology, & political beliefs’ and ‘lobbyists & pressure groups’ should become relevant in the ‘negotiation’ stage only, after the ‘evidence’ stage has been terminated. Based on all previously summarized information, stakeholders - each from their own perspective - have the freedom to discuss, argument and reach agreement.
8. Media attention is not controllable. However, a more systematic application of the procedures above is likely to prevent policy makers to move prematurely from the evidence towards the negotiation stage when feeling pressured by the media (aorta endoprosthesis, oseltamivir, Alzheimer medication, trastuzumab). Minimize pressure from suppliers (aorta endoprosthesis) through normative regulation and make the financial and non financial compensation to care providers and patient organizations public.
9. Finally, periodically review reimbursement decisions, taking into account new evidence that becomes available (Alzheimer medication). However, base the decision to reevaluate on formalized criteria. This will prevent that a care intervention for which reimbursement has been turned down, is reentered repeatedly into the process to let it pass (hadron therapy).

The findings of our study should be interpreted with caution. Being qualitative in nature, they cannot be generalized towards all reimbursement decisions. Future research should examine whether the evidence based policy framework can find similar interactions in other cases. Equal attention should be given to cases in which reimbursement was declined. We interviewed a number of key decision makers only, and not all stakeholders involved. We did not attempt to reach further saturation, because the analysis across cases already revealed a sufficient number of recurring patterns. Since we used a conceptualized framework to detect themes, this might have prevented us from identifying other influential factors. However, all respondents confirmed that our framework provided a sufficient degree of coverage of how each case evolved.

Our findings showed that respondents sometimes disagreed with each other or made statements that were contradictory to what we know to be evidence from the literature (e.g. was the new treatment cost-effective or not; and was this issue taken into account or not). Such inconsistencies are likely due to a mixture of inconsistencies in the evidence itself, selective information use from a personal stakeholder perspective, the possibility that one was not aware of certain facts at the time of decision making and/or recall bias several years after a decision was made.

Finally, we studied reimbursement policy in Belgium only. As reported above, our findings correspond well with general theories and findings in other health systems. There is no reason to assume that components of the framework as a whole will impact reimbursement decision making differently in other developed countries. Many health systems make use of a similar centralized procedure as the one central to the study. Other countries, such as the United States of America, decentralize the decision making to individual insurance agencies. The same factors are likely to be at play, although the degree of negotiation power will be different in such a context. Some findings, such as the impact of cost to patient, the impact of cultural differences and the impact of decision making jurisdictions within a health system are also likely context dependent.

## Conclusions

Reimbursement decisions for new drugs, implants and other health care interventions in Belgium are the outcome of a process of interplay between several factors. Clinical and health economic evidence played a role in many of the decisions, but was often not the predominant factor in the decision made. Based on the analysis of six cases, we formulated nine recommendations, aimed at policy makers in Belgium and other countries making use of payer-provider reimbursement councils, that are expected to reconcile the ‘evidence’ and the ‘negotiation’ stage of health policy decision making in a more systematic and balanced way, without hurting the fundamentals of democratic debate. This may be one of many instruments to cope with the increasing budgetary pressure in health care, by separating real, worthwhile innovations from additional ‘waste’. Future research should focus on the relative importance of influential factors on decision making. A mixed methods approach, adding quantitative to qualitative information, is recommended.
